# The new era of AML therapy: current standards and emerging targets

**DOI:** 10.1007/s10147-026-03038-7

**Published:** 2026-04-17

**Authors:** Naoko Hosono

**Affiliations:** Department of Hematology and Oncology, University of Fukui, 23-3, Shimoaizuki, Matsuoka, Eiheiji, Fukui 910-1193 Japan

**Keywords:** AML, Actionable mutation, FLT3, IDH1/2, Menin, Hypomethylating agent

## Abstract

Over the past decade, AML therapy has evolved from the uniform “7 + 3” regimen toward a personalized approach. This transition integrates molecular genetic profiles with clinical fitness, driven by the genomic elucidation of AML and the development of selective targeted agents. Key advancements include the integration of FLT3 inhibitors into intensive chemotherapy and the emergence of venetoclax. When combined with hypomethylating agents, venetoclax has redefined the standard of care for older or unfit patients. Furthermore, IDH1/2 inhibitors and menin inhibitors have provided potent options for molecular subsets defined by *IDH1/2* mutations and *KMT2A* rearrangements or *NPM1* mutations, respectively. Innovations such as the liposomal formulation CPX-351 and oral formulations of CC-486 and oral decitabine/cedazuridine have further optimized treatment delivery and improved patient quality of life. Despite these breakthroughs, intrinsic clonal heterogeneity and drug-resistant mutations, particularly *TP53* mutation, remain significant challenges. Current research is actively exploring next-generation inhibitors, antibody–drug conjugates, and cellular immunotherapies such as BiTEs and CAR-T cells. This review summarizes the recent pharmacological evolution in AML and discusses how these emerging therapies bring us closer to the ultimate goal of achieving a definitive cure.

## Introduction

Acute myeloid leukemia (AML) is a clonal hematologic malignancy characterized by the neoplastic proliferation of myeloid progenitors. Predominantly affecting the elderly, the median age at diagnosis is 68 years. Since the 1980 s, the standard “7 + 3” induction regime, combining cytarabine and anthracycline, followed by consolidation therapy or allogeneic stem cell transplantation, has aimed at leukemic cell eradication. However, while half of patients achieve remission, a high incidence of relapse results in a disappointing 5 year overall survival rate of 35–40% [1, 2]. Prognosis remains particularly poor for patients with high-risk cytogenetics or those too frail for intensive chemotherapy.

Following the Human Genome Project’s elucidation of leukemic molecular genetics in the early 2000 s, targeted therapies have rapidly advanced. Specifically, molecular targeted agents such as FLT3 and BCL2 inhibitors, as well as hypomethylating agents (HMAs), have been extensively investigated in clinical trials. Since the late 2010 s, combination regimens incorporating these agents have transformed the AML landscape, significantly improving remission rates and survival, even in elderly or high-risk populations. This pharmacological paradigm continues to evolve. This article reviews the dramatic evolution of AML pharmacotherapy over the past decade and discusses novel agents expected in the future.

## Therapeutic strategies for AML

AML treatment strategies are primarily determined by the cytogenetic abnormalities and molecular genetic profiles of the leukemic cells, as well as the patient's performance status (intensive chemotherapy eligibility). For patients deemed unfit for intensive chemotherapy due to comorbidities or organ dysfunction, the therapeutic strategy incorporates a backbone of hypomethylating agents (HMAs) combined with novel agents, notably venetoclax. When specific molecular targets, such as *FLT3*, *IDH1/2*, *KMT2A* rearrangements, or *NPM1,* are identified, corresponding targeted therapies are considered within the treatment algorithm. Prognostic stratification follows the ELN 2022 risk classification for intensive chemotherapy [3] and ELN 2024 for less-intensive therapies [4]. Furthermore, the optimal strategy is determined through a comprehensive assessment that integrates patient preferences and social support, facilitating shared decision-making. For potential allogeneic hematopoietic stem cell transplantation (allo-HSCT) candidates, a systematic management framework evaluating transplant eligibility from the time of diagnosis is highly recommended.

## Novel therapeutic agents for AML

Individualized therapy in AML is driven by an enhanced understanding of molecular pathogenesis and the resulting targeted agents. Historically, optimizing cytotoxic regimens yielded limited improvements for poor-prognosis patients. A major turning point was the 2013 The Cancer Genome Atlas (TCGA) report [5], which detailed the genomic landscape of AML. This analysis revealed that only three genes, *FLT3*, *NPM1*, and *DNMT3A,* exhibit high-frequency mutations (> 10%), while most others occur in < 5% of cases [5]. Building on this, research identified mutations and co-mutational patterns associated with chemotherapy resistance [6], accelerating the development of selective inhibitors for *FLT3*, *IDH1/2*, and menin. Additionally, to overcome the overexpression of anti-apoptotic proteins (e.g., Bcl-2), the BCL2 inhibitor venetoclax (VEN) was introduced, becoming a cornerstone of current AML therapy.

Therapeutic strategies have expanded beyond molecular-targeted agents. Innovations include HMAs targeting epigenetic regulation and CPX-351, which utilizes a liposomal drug delivery system (DDS) to efficiently deliver daunorubicin and cytarabine to leukemic cells. Due to their distinct mechanisms, these agents have demonstrated efficacy against secondary and high-risk AML, which were previously resistant to conventional chemotherapy. As shown in Table 1, numerous AML agents have been approved in the United States, Europe, and Japan since 2018. To provide a comprehensive overview of this changing landscape, this review explores the recently approved agents and examines the next generation of therapies that are redefining the future of AML treatment.

**Table 1:** Novel AML therapeutic agents approved in recent years

Agents	Mode of Action	U.S	EMA	JPN
Midstaurin	FLT3 inhibitor	2017	2017	-
Gilteritinib	FLT3 inhibitor	2018	2019	2018
Quziartinib	FLT3 inhibitor	2023	2023	2019
Venetoclax	Bcl-2 inhibitor	2018	2021	2021
Glasdegib	SMO inhibitor	2018	2020	-
Ivosidenib	IDH1 inhibitor	2018	2023	2025
Olutasidenib	IDH1 inhibitor	2022	-	-
Enasidenib	IHD2 inhibitor	2017	-	-
Revumenib	Menin inhibitor	2024	-	-
Ziftomenib	Menin inhibitor	2025	-	-
Azacitidine	Hypomethylating agents	(MDS)	2015	2021
CC-486 (oral)	Hypomethylating agents	2020	2021	-
Decitabine	Hypomethylating agents	(MDS)	2016	-
Decitabine/cedazuridine (oral)	Hypomethylating agents	(MDS)	2023	-
Gemtuzumab Ozogamicin	Calicheamicin-conjugated humanized anti-CD33 monoclonal antibody	2017	2018	2005
CPX-351	Liposomal Ara-C/DNR	2017	2018	2024

## Molecular targeted therapies

### 1) FLT3 inhibitors

*FLT3* mutations occur in 25–30% of AML cases, including internal tandem duplication (ITD) (approximately 25%) and tyrosine kinase domain (TKD) mutations (7–10%) [4]. Previously associated with high relapse and poor prognosis, *FLT3*-ITD positive AML was reclassified into the intermediate group in the 2022 ELN guidelines due to the success of FLT3 inhibitors (Table 1). Inhibitors are classified into first-generation (midostaurin, sorafenib) and second-generation (gilteritinib, quizartinib) agents. Mechanistically, Type 1 inhibitors (midostaurin, gilteritinib) target both ITD and TKD mutations, while Type 2 inhibitors (quizaritinib, sorafenib) primarily target ITD. Combining “7 + 3” chemotherapy with FLT3 inhibitors is now a standard of care for newly diagnosed AML. In the RATIFY trial, adding midostaurin significantly improved median overall survival (OS) to 74.7 months versus 25.6 months with placebo [7]. Similarly, the QuANTUM-First trial showed that quizartinib plus chemotherapy improved median OS (31.9 vs. 15.1 months; HR 0.776) [8], with benefits maintained through long-term follow-up and maintenance therapy.

For unfit patients, triplet regimens adding an FLT3 inhibitor to azacitidine (AZA) and VEN are under investigation (NCT04140487, NCT04687761). Despite these advances, resistance remains a challenge. Type 2 inhibitors often trigger secondary TKD mutations (e.g., D835), while Type 1 inhibitors may encounter RAS/MAPK activation or the F691L gatekeeper mutation [9]. To address F691L-mediated resistance, novel agents, such as pexidartinib [10] and FF-10101–01 [11] are currently in Phase 1/2 trials (NCT01349049, NCT03194685).

### 2) IDH1/2 inhibitors

Isocitrate dehydrogenase (IDH) is a key metabolic enzyme. In AML, *IDH1* (7–15%) and *IDH2* (5–15%) mutations [5, 12, 13] trigger the production of the oncometabolite 2-hydroxyglutarate (2-HG), leading to epigenetic dysregulation and differentiation arrest [14, 15]. IDH1/2 inhibitors potently suppress 2-HG production by mutated IDH1/2, thereby reversing epigenetic dysregulation and acting as differentiation-inducing agents. This process overcomes the differentiation arrest, allowing leukemic cells to mature leukocyte.

### i) IDH1 inhibitor

Ivosidenib (IVO) is a first-in-class oral inhibitor that selectively targets mutant IDH1. In July 2018, it was FDA-approved for the treatment of R/R *IDH1*-mutated AML. In the Phase 3 AGILE trial for unfit, newly diagnosed *IDH1*-mutated AML, IVO + AZA demonstrated superior clinical efficacy over placebo + AZA, significantly prolonging median OS (24.0 vs. 7.9 months, P = 0.001) [15–17]. With a markedly higher CR rate (47.2% vs. 14.9%), IVO + AZA has consequently become a standard first-line therapy globally. However, resistance can emerge via *IDH1* S280F mutations or "isoform switching" to *IDH2* [18, 19]. Olutasidenib, a novel IDH1 inhibitor effective against S280F, demonstrated a 35% CR/CRh rate and a durable median response of 25.9 months in R/R IDH1-mutated AML [20]. Notably, olutasidenib maintains efficacy even after prior VEN exposure, making it a promising therapeutic option for patients who fail AZA + VEN combination therapy.

### ii) IDH2 inhibitor

Enasidenib, an IDH2 inhibitor, was granted FDA approval in August 2017. In a Phase 3 trial for elderly patients with R/R *IDH2*-mutated AML, enasidenib monotherapy improved EFS over salvage chemotherapy (4.9 vs. 2.6 months) but did not significantly prolong OS (6.5 vs. 6.2 months) [21]. In newly diagnosed unfit patients, enasidenib with AZA significantly improved the ORR (74% vs. 36%, P = 0.0003) compared to AZA alone, although OS differences were likely confounded by subsequent salvage therapies in the control arm [22].

### 3) Menin inhibitors

Menin, a nuclear scaffold protein encoded by *MEN1*, regulates gene expression by interacting with transcription factors and chromatin regulators [23]. In leukemias with *KMT2A* rearrangements (KMT2Ar) or *NPM1* mutations, menin binds to the KMT2A complex to drive the aberrant expression of *HOXA* cluster genes and *MEIS1*, promoting leukemogenesis [24–27]. Based on these findings, menin inhibitors are being developed as promising targeted therapies for AML with KMT2Ar or *NPM1* mutations. In 2024, revumenib (SNDX-5613) was approved by the FDA as the first-in-class menin inhibitor. Currently, numerous clinical trials are underway for other menin inhibitors, including bleximenib (JNJ-75276617), enzominib (DS-1594), ziftomenib (KO-539), and BMF-219, which are anticipated to further improve clinical outcomes.

Revumenib (SNDX-5613) was evaluated in the Phase 1/2 AUGMENT-101 trial (NCT04065399) involving patients with R/R acute leukemia with KMT2Ar or *NPM1* mutations. In this heavily pretreated population (n = 68), the CR/CRh rate was 30% [28]. Subsequent analysis of the Phase 2 cohort (n = 57) demonstrated the ORR of 63.2% and the CR/CRh rate of 22.8%, confirming its potent clinical activity [29]. Ziftomenib (KO-539) demonstrated promising activity in the Phase 1/2 KOMET-001 trial, with a 26% CRc rate in *NPM1*-mutated AML [30]. Enzomenib (DSP-5336) is also being evaluated (NCT04988555); interim data showed CR/CRh rates of 22.7% (KMT2Ar) and 23.1% (*NPM1*), with a rapid median response time of 1.0 month [31]. More recently, inhibitor-naive KMT2Ar patients treated at the recommended phase 2 dose achieved a 73.3% ORR and 40% CR/CRh [32]. Similarly, bleximenib (JNJ-75276617) demonstrated an ORR of 50% with a median DOR of 6.4 months in Phase 1 trials [33], with triplet regimens (bleximenib, AZA + VEN) now under investigation.

Regarding safety, differentiation syndrome (DS) is a distinctive adverse event, occurring in 16–25% of cases. Consistent with the clinical course of differentiation-induction therapy, menin inhibitors promote the differentiation of leukemic blasts, leading to the recovery of normal hematopoiesis and the clearance of genetic aberrations. Beyond KMT2Ar and *NPM1* mutations, the menin-KMT2A interaction drives leukemogenesis in other *HOXA/MEIS1*-overexpressing leukemias, including *NUP98* rearrangements [34], *UBTF* tandem duplications [35], and *CEBPA* mutations [36]. Consequently, further expansion of therapeutic indications is anticipated.

### 4) BCL2 inhibitors

BCL2, an anti-apoptotic protein overexpressed in AML and leukemia stem cells (LSCs), is a key factor driving resistance to conventional chemotherapy. Venetoclax (VEN) was developed as a highly selective, first-in-class BCL2 inhibitor that specifically targets the Bcl-2 homology (BH) domains [37]. For treatment-naive patients who are ineligible for intensive chemotherapy, the combination of AZA + VEN has become the widely adopted standard of care. The efficacy of this combination was demonstrated in the Phase 3 VIALE-A trial (NCT02993523), which showed a significant improvement in median OS: 14.7 months in the AZA + VEN group compared to 9.6 months in the AZA + placebo group (HR = 0.66; 95% CI 0.52–0.85, p < 0.001) [38]. Similarly, the VIALE-C trial evaluated VEN plus low-dose cytarabine (LDAC); an updated analysis confirmed a significant survival benefit with a median OS of 8.4 months (HR 0.70, P = 0.04) [39]. Building on its success in the frontline setting, the combination of VEN and HMAs has also emerged as a potent salvage therapy for R/R AML [40]. Currently, an ongoing Phase 2 trial (NCT04801797) is comparing intensive chemotherapy versus VEN + AZA in treatment-naive patients to further define its role.

Furthermore, sonrotoclax (BGB-11417) is being developed as a second-generation BCL2 inhibitor with potentially higher selectivity and potency. In a Phase 1b/2 trial (NCT04771130), sonrotoclax + AZA demonstrated an acceptable safety profile and high efficacy, with an ORR of 74.7% and a CR of 50.6% [41]. As a potential next-generation option, sonrotoclax may offer enhanced clinical outcomes over current inhibitors.

## Hypomethylating agents (HMAs)

AML is a biologically heterogeneous disease in which epigenetic dysregulation, specifically DNA hypermethylation, plays a central role in pathogenesis and prognosis [42]. Because the hypermethylation of tumor suppressor gene promoters drives leukemogenesis, DNA methyltransferase inhibitors (azacitidine and decitabine) were first established for myelodysplastic syndromes (MDS). Subsequently, their clinical utility has been extensively demonstrated in AML, where they now serve as a foundational therapeutic component.

### 1) Azacitidine (AZA)

In the Phase 3 AZA-AML-001 trial, 488 elderly patients were randomized to AZA or conventional care regimens (CCR) [43]. Although the AZA group showed a trend toward prolonged median OS (10.4 vs. 6.5 months; HR 0.85, P = 0.1087) without reaching the threshold for statistical significance, Europe and Japan granted approval based on survival benefits observed in sensitivity and domestic analyses [44]. Conversely, the US FDA withheld approval for AZA monotherapy in this setting due to the lack of a statistically significant OS improvement.

### 2) Decitabine (DAC)

Decitabine (DAC), another primary DNA methyltransferase inhibitor, has shown comparable clinical utility. In the Phase 3 DACO-016 trial (n = 485), DAC improved median OS compared to the control group (7.7 vs. 5.0 months; HR 0.82, P = 0.037) [45]. Consequently, the EMA approved DAC for elderly AML, though the FDA declined approval for monotherapy following updated analyses. In Europe, the Phase 3 EORTC-1301/AML21 trial compared DAC with the standard “7 + 3” regimen in fit patients aged 60 and older [46]. While the 4 year survival rates were comparable between the DAC and 7 + 3 groups (26% vs. 30%; HR 1.04, p = 0.68), DAC demonstrated a superior safety profile with significantly lower rates of infection and gastrointestinal toxicity, a clinical benefit further supported by recent 6-year follow-up data confirming maintained long-term efficacy (23.7% vs. 25.5%, respectively) [47].

### 3) Oral azacitidine (CC-486)

In addition to injectable agents, oral formulations of HMAs have also been developed. CC-486 is an oral formulation of AZA with pharmacokinetic and pharmacodynamic profiles distinct from its injectable counterpart; it is primarily metabolized in the liver and not excreted renally [48]. The Phase 3 QUAZAR AML-001 trial evaluated CC-486 as maintenance therapy for patients in first complete remission (CR1) following intensive chemotherapy [49]. In this study, CC-486 (n = 238) significantly improved median OS compared to the placebo group (24.7 vs. 14.8 months, P < 0.001). Based on these data, CC-486 was approved in the US (2020) and Europe (2021) specifically for AML maintenance therapy. In Japan, a domestic Phase 2 trial (jRCT2011210063) has also been completed to support its clinical utility.

### 4) Oral decitabine/cedazuridine

In addition to CC-486, oral decitabine/cedazuridine (ASTX727) is a fixed-dose combination designed to prevent DAC degradation. Since DAC is rapidly metabolized by cytidine deaminase (CDA) in the gastrointestinal tract and liver, the addition of cedazuridine, a CDA inhibitor, enables effective oral delivery. The Phase 3 ASCERTAIN trial confirmed that ASTX727 provides pharmacokinetic exposure (AUC) nearly equivalent to intravenous DAC [50]. Building on this, the ASCERTAIN-V trial is evaluating an all-oral regimen of ASTX727 plus venetoclax (VEN). Latest data show promising efficacy, with the CR rate 46.5%, the CR/CRi rate of 63.4%, and a median OS of 15.5 months [51]. By providing treatment intensity comparable to injectable decitabine without the requirement for daily clinical visits, this oral regimen facilitates improved QOL and treatment adherence, particularly in the elderly population.

## Liposomal cytarabine and daunorubicin (CPX-351)

CPX-351 is a liposomal formulation of cytarabine and daunorubicin at a fixed 5:1 molar ratio [52]. This encapsulation maintains the optimal ratio in the blood and bone marrow for over 24 h, enhancing drug delivery. Consequently, CPX-351 has shown significant efficacy in therapy-related AML (tAML) and secondary AML (sAML) [53]. In a Phase 3 trial of elderly patients (aged 60–75) with high-risk AML, CPX-351 significantly improved median OS compared to the standard “7 + 3” regimen (9.56 vs. 5.95 months; HR 0.69) and increased the CR/CRi rate (47.7% vs. 33.3%) [54]. Notably, 34% of the CPX-351 group proceeded to hematopoietic cell transplantation (HCT), highlighting its utility as an effective bridge to transplant. Based on these data, CPX-351 was approved for high-risk AML in the US, Europe, and Japan.

## Agents under development

**Fig. 1:**
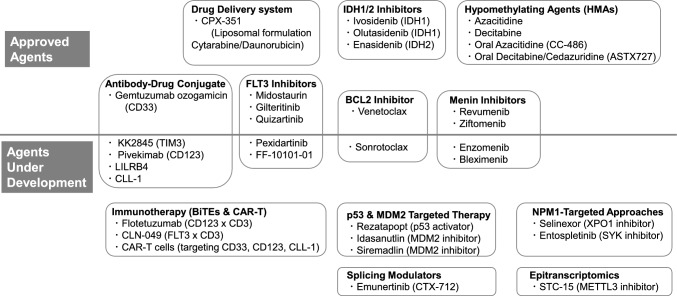
The landscape of current and emerging therapeutics in acute myeloid leukemia (AML)

While the current therapeutic landscape has been significantly advanced by these approved agents, the drug development pipeline continues to expand with novel targets and second-generation inhibitors. An overview of these emerging agents, currently in clinical trials or under advanced development, is summarized in Table 2 and Fig. 1.

**Table 2:** Investigational agents in clinical trials for AML

Drug	Function	Disease	Phase	NCT Number
Vosaroxin (SNS-595)	anticancer quinolone (topoisomerase II inhibition)	R/R AML	3	NCT01191801
Sapacitabine (CYC682)	nucleoside analog (DNA strand breaks)	ND-AML (elderly)	3	NCT01303796
Lisaftoclax (APG-2575)	BCL-2 inhibitor	ND-AML (unfit)	3	NCT06389292
Ruserontinib (SKLB1028)	multikinase inhibitor	R/R FLT3-mutated AML	3	NCT04716114
Clifutinib (HEC73543)	FLT3 inhibitor (2nd generation)	R/R FLT3-mutated AML	3	NCT05586074
Radgocitabine (DFP-10917)	deoxycytidine nucleoside analog	R/R AML	3	NCT03926624
Entospletinib (GS-9973)	Syk inhibitor	ND-NPM1 mutated AML	3	NCT05020665
Devimistat (CPI-613)	Metabolic inhibitor (TCA cycle)	R/R AML	3	NCT03504410
Bleximenib*	Menin inhibitor	Acute leukemia (KMT2Ar or NPM1 mut)	3	NCT06852222
Amonafide L-Malate	Topoisomerase II inhibitor	untreated sAML	2	NCT00273884
Selumetinib	MEK1 and MEK2 inhibitor	R/R AML	2	NCT00588809
MP0533	Tetra-specific T-cell engager (CD33, CD123, CD70, CD3)	R/R AML/MDS	1/2a	NCT05673057
Bexmarilimab (FP-1305)	Clever-1 Antibody	R/R AML/MDS	1/2	NCT05428969
APL-4098	GCN2 (EIF2AK4) inhibitor	R/R AML/MDS	1/2	NCT06372717
Enzomenib (DSP-5336) *	Menin inhibitor	R/R Acute leukemia (KMT2Ar or NPM1 mut)	1/2	NCT04988555
Tuspetinib (HM43239)	Multikinase inhibitor (SYK, FLT3, JAK1/2, RSK2)	R/R AML	1/2	NCT03850574
LBS-007	CDC7 inhibitor	R/R Acute leukemia	1/2	NCT05756322
Pacritinib (SB1518)	JAK2/FLT3 inhibitor	Myeloid malignancy	1/2	NCT00719836
S 64315	Mcl-1 inhibitor	R/R AML	1	NCT03672695
UCART123v1.2	CD123 Universal Chimeric Antigen Receptor T-cell	R/R AML	1	NCT03190278
Ilorasertib (ABT-348)	Multikinase inhibitor (Aurora kinase family)	R/R AML	1	NCT01110473
IGN523	CD98 monoclonal antibody	R/R AML	1	NCT02040506
Ocifisertib (CFI-400945)	Polo-like Kinase 4 inhibitor	R/R AML	1	NCT03187288
JNJ-63709178	Bispecific Antibody (CD123 × CD3)	R/R AML	1	NCT02715011
PRT543	PRMT5 inhibitor	R/R AML and others	1	NCT03886831
Iadademstat (ORY-1001)	LSD1/KDM1A inhibitor	R/R FLT3-mutated AML	1	NCT05546580
BMS-986497 (ORM-6151)	Anti-CD33 GSPT1 Molecular Glue Degrade	R/R AML/MDS	1	NCT06419634
GLB-001	Casein Kinase 1 alpha Molecular Glue Degrader	R/R AML/MDS	1	NCT06146257
Denfivontinib (SKI-G-801)	FLT3/AXL inhibitor	R/R AML	1	NCT03564288
IO-202	LILRB4 monoclonal antibody	R/R AML, CMML	1	NCT04372433
BP1002	Liposomal Bcl-2 Antisense Oligodeoxynucleotide	R/R Acute leukemia	1	NCT05190471

*Clinical trials for these agents are also being conducted in Japan

### 1) Antibody–drug conjugates (ADCs)

Antibody–drug conjugates (ADCs) deliver cytotoxic payloads directly to leukemia cells by targeting specific surface antigens, thereby minimizing off-target toxicity [55]. Gemtuzumab ozogamicin (GO), a CD33-targeted ADC, is widely used in clinical practice. Its addition to the standard “7 + 3” regimen has significantly improved survival outcomes, particularly in favorable-risk cases such as core-binding factor (CBF) AML [56]. Due to hematologic toxicity from CD33 expression on normal progenitors, next-generation ADCs with enhanced leukemic selectivity are currently under development. Clinical trials are underway targeting antigens such as TIM3 (KK2845), CD123 (Pivekimab), LILRB4, and CLL-1, aiming to optimize efficacy while reducing treatment-related toxicity [57–60].

### 2) Cellular immunotherapy: BiTE and CAR-T

Immunotherapy, encompassing bispecific antibodies and adoptive cell therapies, represents a rapidly evolving frontier aimed at engaging the patient's immune system to target AML. Bispecific T-cell engagers (BiTEs) bridge leukemia cell antigens with CD3 on T cells, inducing direct T-cell-mediated cytotoxicity. Flotetuzumab, a CD123 × CD3 DART antibody, demonstrated an ORR of 30% in R/R AML, with notably higher activity (47%) in *TP53*-mutated cases [61, 62]. Vibecotamab (CD123 × CD3) showed an ORR of 14% with favorable tolerability [63, 64]. For AMG 330 (CD33 × CD3), a Phase 1 study reported CR/MLFS in 8 of 60 patients, with significant blast reduction in 37% of non-responders [65]. Additionally, CLN-049 (FLT3 × CD3) is showing encouraging preliminary efficacy, with a CRc rate of 30% and an acceptable safety profile [66].

Chimeric antigen receptor (CAR) T-cell therapy is a form of cellular immunotherapy that utilizes genetically modified T cells engineered to express receptors that specifically recognize antigens on the surface of cancer cells. While this therapy has achieved dramatic success in B-cell lymphoid malignancies, including acute lymphoblastic leukemia (ALL), its application to AML is also highly anticipated. Currently, clinical trials for CAR-T cell therapies targeting antigens such as CD33 (NCT03971799), CD123 (NCT04318678), and CLL-1 (NCT03222674) are underway.

### 3) Emerging novel targets

#### I) p53 Activators (rezatapopt: PC14586)

*TP53* mutations are among the most challenging genetic abnormalities in AML, consistently associated with treatment resistance and poor prognosis. While broad-spectrum p53 activators like APR-246 failed to demonstrate sufficient efficacy across various mutations in Phase 3 trials, the field is now shifting toward more targeted approaches. Rezatapopt (PC14586) represents this next generation of precision therapy; it is a mutation-specific agent designed to restore functional p53 activity by normalizing the structural conformation specifically of the *TP53* Y220C mutation. Given that monotherapy may provide limited apoptotic induction [67], focus has shifted to evaluating its efficacy in combination with AZA and VEN in ongoing clinical trials (NCT06616636).

#### ii) MDM2 inhibitors

Beyond direct TP53 modulation, the MDM2-p53 axis represents another critical target for restoring p53 function. MDM2 is an E3 ubiquitin ligase and the primary negative regulator responsible for p53 degradation. MDM2 inhibitors have been developed to reactivate p53 and induce cell death by blocking the interaction between MDM2 and p53. In a Phase I trial (NCT02670044) evaluating the combination of the MDM2 inhibitor idasanutlin (RG7388) and VEN for R/R AML, the CRc rate was 26% (including 20% in patients with TP53 mutations) [68]. Several other MDM2 inhibitors are currently under clinical investigation: KT-253 is being evaluated as a monotherapy (NCT05775406), while alrizomadlin (APG-115) is being tested in a doublet combination with VEN (NCT03940352). Furthermore, siremadlin (HDM201) is undergoing trials in a triplet combination with VEN and AZA (NCT05155709) to optimize both efficacy and safety.

#### iii) Agents targeting *NPM1* mutations (XPO1 inhibitors and SYK inhibitors)

*NPM1* mutations, found in approximately 30% of AML cases, represent a critical therapeutic target with several emerging strategies beyond menin inhibition. These novel approaches focus on disrupting the aberrant transport and signaling pathways associated with mutant NPM1 (NPM1c) [69, 70]. One such target is the nuclear export protein XPO1 (exportin-1), which interacts with NPM1c to drive HOX gene overexpression and impair differentiation. Selinexor (KPT-330), an XPO1 inhibitor, is currently undergoing clinical evaluation (NCT01607892). Furthermore, *NPM1*-mutated AML exhibits a distinct dependency on the SYK (spleen tyrosine kinase) signaling pathway via HOXA9/MEIS1 activation. The SYK (spleen tyrosine kinase) inhibitor entospletinib is now being investigated in a Phase 3 trial (NCT05020665) in combination with intensive induction chemotherapy for patients with newly diagnosed *NPM1*-mutated AML, and the outcomes of this study are expected to further clarify its clinical role.

#### iv) Splicing modulators

Splicing abnormalities play a central role in the development of AML with myelodysplasia-related changes (AML-MRC), making the splicing machinery an attractive therapeutic target. Emunertinib (CTX-712) is a first-in-class, selective oral CLK inhibitor that regulates RNA splicing. In a Phase 1 trial for MDS and R/R AML, CTX-712 showed encouraging activity, with 5 of 8 AML patients achieving CR/CRi [71]; a Phase 2 trial is ongoing (NCT05732103). Additionally, H3B-8800, a splicing modulator directly targeting SF3B1, is currently being evaluated in a Phase 1 trial (NCT02841540) for *SF3B1*-mutated MDS, AML, and CMML.

#### v) METTL3 inhibitors

Emerging insights into epitranscriptomics have identified RNA modification as a novel regulatory layer in leukemogenesis. METTL3 (methyltransferase-like 3), an mRNA "writer" protein, regulates mRNA stability and translation by mediating *N*^6^-methyladenosine (*m*^6^*A*) methylation. In AML, METTL3 overexpression promotes the translation of oncogenes such as *MYC* and *BCL2*, thereby maintaining the self-renewal capacity of leukemic cells and impairing differentiation [72]. Targeting this mechanism is currently under investigation as a novel therapeutic approach. STC-15, a first-in-class oral METTL3 inhibitor, is being evaluated in an ongoing Phase 1 trial (NCT05584111) for advanced solid tumors and hematologic neoplasms.

## Conclusion

The therapeutic landscape of AML has undergone a paradigm shift, as the emergence of novel targeted agents has redefined treatment strategies. Moving away from the conventional "one-size-fits-all" approach, modern management is now tailored based on both leukemia-specific molecular profiles and patient-specific factors, including organ function, performance status, and treatment preferences. For "fit" patients, the addition of targeted therapies to intensive chemotherapy or the use of liposomal formulations has optimized outcomes, with risk-adapted allogeneic transplantation remaining the primary curative goal. Conversely, for “unfit” or elderly patients, BCL-2 inhibitor-based regimens have extended survival from months to years, occasionally serving as a bridge to transplant.

Despite these advances, the clonal heterogeneity of AML presents a persistent risk of relapse through the selection of drug-resistant clones. Overall survival still lags behind the remarkable outcomes observed in chronic myeloid leukemia (CML), indicating that significant unmet needs remain. Nevertheless, the ongoing clinical validation of the diverse emerging therapies discussed in this review brings us closer than ever to overcoming these barriers. By expanding therapeutic options and refining precision approaches, we move toward achieving the ultimate goal: a definitive cure for AML.

This schematic summarizes the evolving therapeutic armamentarium for AML, categorized by regulatory status and mechanism of action: Approved Agents (upper panel) and Agents Under Development (lower panel).
